# Effects of Black Garlic Extract and Nanoemulsion on the Deoxy Corticosterone Acetate-Salt Induced Hypertension and Its Associated Mild Cognitive Impairment in Rats

**DOI:** 10.3390/antiox10101611

**Published:** 2021-10-13

**Authors:** Chun-Yu Chen, Tsung-Yu Tsai, Bing-Huei Chen

**Affiliations:** 1 Department of Food Science, Fu Jen Catholic University, New Taipei City 24205, Taiwan; pack24578@yahoo.com.tw (C.-Y.C.); tytsai@mail.fju.edu.tw (T.-Y.T.); 2 Department of Nutrition, China Medical University, Taichung 40401, Taiwan

**Keywords:** black garlic nanoemulsion, organosulfur compounds, phenolic acids and flavonoids, Morris water maze test, systolic blood pressure, rats

## Abstract

Organosulfur compounds, phenolic acids and flavonoids in raw and black garlic were determined, and followed by preparation of black garlic nanoemulsion for studying their effects on deoxycorticosterone acetate-salt-induced hypertension and associated mild cognitive impairment in rats. Three organosulfur compounds, including diallyl sulfide (87.8 μg/g), diallyl disulfide (203.9 μg/g) and diallyl trisulfide (282.6 μg/g) were detected in black garlic by GC-MS, while gallic acid (19.19 μg/g), p-coumaric acid (27.03 μg/g) and quercetin (22.77 μg/g) were detected by UPLC-MS/MS. High doses of both black garlic extract and nanoemulsion prepared using Tween-80, glycerol, grapeseed oil and water could decrease systolic blood pressure through the elevation of bradykinin and nitric oxide levels as well as diminish aldosterone and angiotensin II levels in rats. In Morris water maze test, they could significantly decrease escape latency and swimming distance and increase the time spent in the target quadrant, accompanied by a decline of acetylcholinesterase activity and malondialdehyde level in the hippocampus as well as a rise in glutathione level and activities of superoxide dismutase, catalase and glutathione peroxidase. In addition, the levels of tumor necrosis factor, interleukin-6 and interleukin-1β were reduced. Effects of lowering blood pressure and improving learning/memory ability in rats followed the order: lisinopril > black garlic nanoemulsion > black garlic extract.

## 1. Introduction

Garlic (*Allium sativum*) is a popular spice used in many kinds of foods during cooking. Many studies have demonstrated that the consumption of garlic can be effective against chronic diseases such as diabetes and cancer [1,2], which can be attributed to the presence of functional components such as allicin and S-methyl cysteine sulfoxide. However, due to the presence of its pungent odor, the application of garlic to the food industry has been limited. Thus, in recent years many processing techniques have been developed to remove this pungent odor. For instance, black garlic is a naturally aged product without a pungent odor.

Accordingly, black garlic can be produced from raw garlic under high-temperature and high-humidity conditions. During aging, raw garlic color can turn into black caused by the Maillard reaction and caramelization, accompanied by the conversion of pungent odor compounds to tasteless compounds [3]. In addition, compared to raw garlic, black garlic possessed a stronger antioxidant activity, as evidenced by enhancement of activities of superoxide dismutase (SOD) and catalase, as well as polyphenol level by 13- and 10- and 7-fold, respectively [4]. In addition to organosulfur compounds, the presence of polyphenols in black garlic should also play an important role in biological activity. More specifically, the levels of total phenolic acid and total flavonoid were reported to be 8.20 mg gallic acid equivalent (GAE)/g and 1.92 μg catechin/mg in black garlic, respectively, while in raw garlic, the levels were 0.78 mg GAE/g and 1.40 μg catechin/mg [5,6]. This difference may be accounted for by release of free phenolic acid from complex of phenolic compound–carbohydrate or phenolic compound–protein during garlic aging at high temperature [7]. In addition, more polyphenol compounds can be produced from a non-enzymatic browning reaction during garlic aging [8]. Therefore, the presence of sulfur-containing compounds, phenolic acids and flavonoids in black garlic should be important in preventing chronic disease. Nevertheless, all these bioactive compounds may undergo vaporization or degradation in vivo, resulting in a decrease of biological activity, especially for the highly volatile organosulfur compounds. To remedy this problem, the development of an appropriate technique such as nanoemulsion for encapsulation of bioactive compounds and enhancement of biological activity in vivo is extremely important.

Like garlic, it has been well documented that black garlic possesses many vital biological activities such as liver protection [9], anti-inflammation [10], and anti-cancer [11]. However, the effect of black garlic on the reduction of blood pressure and prevention of neurodegenerative disease using an animal model was less explored.

Dementia, a brain disease that frequently occurs for the elderly, can cause deterioration of cognition ability and memory. According to a report by Alzheimer’s Disease International [12], the dementia population was estimated to be 50 million in 2018 and could reach a record high of 152 million in 2050 worldwide. Many factors have been shown to be associated with cognitive impairment, one of which is hypertension. However, the underlying mechanism remains unclear. It was suggested that the elevated blood pressure could lead to cerebrovascular remodeling [13]. When blood pressure is raised, the vascular wall smooth muscle hypertrophy will occur to decrease lumen diameter, resulting in vascular resistance increase and arteriosclerosis. Meanwhile, it can cause lesions of the aorta and arteriole, as well as white matter and gray matter in the brain, leading to cognitive impairment [13]. The objectives of this study were to use garlic as raw material to process into black garlic and determine bioactive compounds, including organosulfur compounds by GC-MS, as well as phenolic acids and flavonoids in both raw and black garlic by HPLC-MS/MS. Then, both black garlic extracts and nanoemulsions were prepared to study and compare their inhibition effects on deoxycorticosterone acetate-salt-induced hypertension and its associated mild cognitive impairment in rats. A commercial drug lisinopril was also used for comparison.

## 2. Materials and Methods

### 2.1. Reagents

Standards of organic sulfur compounds including diallyl sulfide, diallyl disulfide, diallyl trisulfide, and internal standard isopropyl disulfide were procured from Sigma-Aldrich Co. (St. Louis, MO, USA). Both phenolic acid and flavonoid standards, including gallic acid, p-coumaric acid, and quercetin were also from Sigma-Aldrich Co.

The HPLC-grade solvent acetone was obtained from Merck Co. (Darmstadt, Germany). Deionized water was made using a Milli-Q water purification system from Millipore Co. (Bedford, MA, USA). The QuEChERS kit including extraction powder containing 4 g anhydrous magnesium sulfate plus 1 g anhydrous sodium acetate, and purification powder including 300 mg primary and secondary amine (PSA), 900 mg anhydrous magnesium sulfate plus 300 mg C18EC (Octadecylsiloxane endcapped), ceramic homogenizer and 50-mL centrifuge tube was from Yu-Ho Co. (New Taipei City, Taiwan). Potassium dihydrogen phosphate, sodium chloride, potassium chloride, lisinopril, deoxycorticosterone acetate, 99% ethanol, 1,1,3,3-tetra methoxy propane (TMP), trichloroacetic acid (TCA), and thiobarbituric acid (TBA) were also from Sigma-Aldrich Co.

### 2.2. Instrumentation

Gas chromatograph (GC, model HP6890) coupled with a mass spectrometer (MS, model 5973) was from Agilent Technologies (Santa Clara, CA, USA). The UPLC system coupled with a triple quadrupole tandem mass spectrometer was from Thermo Fisher Scientific Co. (San Jose, CA, USA). Ultrasonicator (model DC400H) was from Hua-Hsiah Scientific Co. (Taipei, Taiwan). The centrifuge (5810R) was from Eppendorf Co. (Hauppauge, NY, USA). The microcentrifuge (Heraeus Fresco 21) was from Thermo Fisher Scientific Co. (San Jose, CA, USA). The nanoparticle size and zeta potential analyzer (SZ-100) was from Horiba Scientific Co. (Kyoto, Japan). The dynamic light scattering instrument was from Brookhaven Instruments Co. (Holtsville, NY, USA). The transmission electron microscope (TEM, model JEM-1400) was from JEOL Co. (Tokyo, Japan). The rotary evaporator (N-1200A) was from Eyela Co. (Tokyo, Japan). The low-temperature circulating water tank (B402L) was from Firstek Co. (Taipei, Taiwan). The blood pressure gauge (BP-2000) was from Visitech System Co. (Apex, NC, USA).

### 2.3. Processing of Garlic into Black Garlic

Raw garlic was obtained from a local farm located at Yuan-Lin county in Taiwan. Its collection for use in this study is approved by Fu Jen Catholic University (New Taipei City, Taiwan) and exempted from any approval from local/national authorities owing to their use only for research purposes. The voucher specimen of raw garlic was deposited in herbarium of TAIwan Forestry Research Institute (TAIF, Taipei, Taiwan) and identified by Dr. Wen-Liang Chiou (TAIF) based on the accession number 04106. Then raw garlic was naturally aged in an incubator with temperature-controlled at 70 °C and relative humidity at 75% for 45 days, after which black garlic was collected for subsequent experiments. This aging condition was selected based on a report by Zhang et al. [14] suggesting that an appropriate aging temperature should be controlled at 70–80 °C with relative humidity at 80%.

### 2.4. Extraction and Purification of Organosulfur Compounds from Raw and Black Garlic by QuEChERS

A QuEChERS method based on Chiu et al. [15] was modified to extract organosulfur compounds from raw and black garlic. Initially, 10 raw and 10 black garlic were each cut into pieces separately, pooled, and one gram was homogenized and poured into a 50-mL centrifuged tube, followed by adding one ceramic homogenizer, 10-mL deionized water and the mixture was shaken again for one min, followed by adding the extraction powder containing 4 g anhydrous magnesium sulfate and 1 g anhydrous sodium acetate, shaking the mixture vigorously for 1 min and centrifuging at 3200 *g* (4 °C) for 10 min. The supernatant (4 mL) was collected and poured into a centrifuged tube containing 900 mg anhydrous magnesium sulfate, 300 mg PSA and 300 mg C18 EC for purification, followed by shaking the mixture for one min, centrifuging at 3200 *g* (4 °C) for 10 min, collecting the supernatant, filtering through a 0.22-μm membrane filter and injecting for GC-MS analysis.

### 2.5. GC-MS Analysis of Organosulfur Compounds in Raw and Black Garlic

A GC-MS method based on Tocmo et al. [16] was modified to analyze organosulfur compounds in raw black garlic. A DM-5MS capillary column (30 m × 0.25 mm ID, 0.25 μm film thickness) was used to separate three organosulfur compounds within 40 min with He as carrier gas and flow rate at 1 mL/min, injector temperature 210 °C, ion source temperature 200 °C, interface temperature 270 °C, injection volume 1 μL and splitless mode with the following temperature programming condition: initial column temperature 50 °C, maintained for 5 min, increased to 210 °C at 5 °C/min and maintained for three min. Both total ion chromatogram (TIC) and selected ion monitoring (SIM) were used for detection of organosulfur compounds with the scanning range from 0–600 *m*/*z* for TIC, while for SIM, the detection was based on elution order of organosulfur compounds with the first group (5–10 min) for detection of diallyl sulfide with *m*/*z* 73, the second group (10–14 min) for isopropyl disulfide (internal standard) with *m*/*z* 108, the third group (14–21 min) for diallyl disulfide with *m*/*z* 81, and the fourth group (21–48 min) for diallyl trisulfide with *m*/*z* 73. In addition, the various organosulfur compounds in raw and black garlic were identified by comparison of retention time and mass spectra of unknown peaks with reference standards, as well as addition of reference standards to sample extract for co-chromatography.

For quantitation, a total of 6 concentrations (1, 5, 10, 15, 20 and 25 μg/mL) were prepared separately for diallyl sulfide, diallyl disulfide and diallyl trisulfide. Then, for each concentration, the internal standard isopropyl disulfide with a fixed concentration at 10 μg/mL was mixed with each standard. The standard calibration curves were obtained by plotting concentration ratio (standard versus internal standard) against peak area ratio (standard versus internal standard), and the regression equation and coefficient of determination (R^2^) were obtained for each standard. Then the various organosulfur compounds in black garlic were quantified using the following formula:

Organosulfur compound (µg/g) = (As/Ai − b) × 1/a × Ci × V × DF × 1/R × 1/Ws

where

As = peak area of organosulfur compounds
Ai = peak area of internal standard
b = intercept of the regression equation
a = slope of the regression equation
Ci = internal standard concentration (μg/mL)
V = extract volume (mL)
DF = dilution factor
R = recovery (%)
Ws = sample weight (g)

### 2.6. Method Validation

A total of 15 concentrations (0.01, 0.02, 0.03, 0.04, 0.05, 0.1, 0.2, 0.3, 0.4, 0.5, 0.6, 0.7, 0.8, 0.9 and 1 μg/mL) were prepared separately for each organosulfur standard and each concentration injected into GC-MS for determination of limit of detection (LOD) based on S/N ≥ 3 and limit of quantitation (LOQ) based on S/N ≥ 10. For the precision study, the intra-day variability was carried out by analyzing organosulfur compounds in black garlic in the morning, afternoon and evening on the same day with three triplicates for a total of 9 analyses. Similarly, the inter-day variability was performed by analyzing organosulfur compounds in black garlic in the morning, afternoon and evening on three different days for a total of 9 analyses. Both standard deviation (SD) and relative standard deviation (RSD, %) were calculated. For the recovery study, two levels of organosulfur standards, including diallyl sulfide (30 and 90 μg), diallyl disulfide (80 and 240 μg) and diallyl trisulfide (100 and 300 μg) were added to black garlic sample separately for extraction, purification and quantitation by GC-MS. The recovery was obtained by using the following formula:

$$\mathrm{Recovery}(\%)=\;\frac{\mathrm{amount}\;\mathrm{of}\;\mathrm{organosulfur}\;\mathrm{compounds}\;\mathrm{found}\;\mathrm{after}\;\mathrm{GC}-\mathrm{original}\;\mathrm{amount}\;\mathrm{by}\;\mathrm{GC}}{\mathrm{amount}\;\mathrm{of}\;\mathrm{organosulfur}\;\mathrm{standards}\;\mathrm{spiked}\;}\times 100$$

### 2.7. Determination of Phenolic Acids and Flavonoids in Raw and Black Garlic

Two methods based on Kim, Kang and Gweon [8] and Kao et al. [17] were modified to determine total phenolic acids and total flavonoids, respectively. Initially, 30 mL of three different proportions of 50%, 70% and 95% ethanol in water were mixed separately with 3 g of raw or black garlic for comparison of extraction efficiency. Then the mixture was sonicated at 60 °C for 1 h and centrifuged at 3200 *g* for 10 min (25 °C). The supernatant was collected, filtered through a 0.6-μm membrane filter, evaporated to dryness, dissolved in 10 mL of 70% ethanol and filtered through a 0.22-μm membrane filter to obtain the extract. Then 30 μL extract was collected and mixed with 120 μL Folin-Ciocalteu reagent, after which the mixture was reacted in the dark for 5 min, followed by adding 600 μL sodium carbonate solution (15%), mixing and reacting in the dark for 1 h and the absorbance was measured at 750 nm. The content of total phenolic acids (expressed as gallic acid equivalent) was calculated based on the standard curve of gallic acid solution (in ethanol), which was obtained by plotting five different concentrations (50, 100, 200, 300 and 400 μg/mL) of gallic acid standard against absorbance.

Likewise, for total flavonoids determination, 500 μL extract was collected and mixed with 30 μL sodium nitrite solution (5%), after which the mixture was left to stand at room temperature for 5 min. Then 60 μL aluminum chloride solution (10%) was added, after which the solution was left to stand for 5 min, followed by adding 300 μL sodium hydroxide solution (1 M) and 200 μL chloroform, centrifuging, collecting the supernatant and measuring absorbance at 510 nm. The content of total flavonoid (expressed as quercetin equivalent) was calculated based on the standard curve of quercetin, which was obtained by plotting 6 different concentrations of quercetin (5, 10, 25, 50, 100, and 200 μg/mL) against absorbance.

For the determination of individual phenolic acids and flavonoids in raw and black garlic, a method based on Kim, Kang and Gweon [8] was modified. A 3-g raw or black garlic sample was mixed with 2.0 g/L butylated hydroxyanisole (BHA) in methanol and 5 mL acetic acid solution (10%). After mixing thoroughly, 15-mL deionized water was added, followed by sonicating for 30 min, adding 60 mL deionized water containing 22 mM EDTA and 2% ascorbic acid, as well as 25 mL sodium hydroxide (10 M). Then the solution was left at 30 °C for 30 min, followed by adding 4 M hydrochloric acid to adjust pH to 2, repeating extraction with 15-mL ethyl acetate three times. All the extracts were pooled, passed through anhydrous sodium sulfate, evaporated to dryness under nitrogen, dissolved in 2 mL methanol and filtered through a 0.22-μm membrane filter for UPLC-MS/MS analysis [8]. Gallic acid, p-coumaric acid and quercetin were separated within 25 min by using an ACQUITY UPLC BEH C18 column (100 mm × 2.1 mm ID, 1.7 μm particle size) with column temperature at 25 °C, flow rate at 0.3 mL/min and a gradient mobile phase of (A) 0.1% formic acid solution and (B) acetonitrile: 95% A and 5% B initially, changed to 85% A and 15% B in 7 min, 70% A and 30% B in 15 min, 100% B in 17 min and maintained for 8 min.

A Dionex Ultimate 3000 model Open Sampler XRS UPLC System coupled with TSQ Quantiva triple quadrupole tandem mass spectrometer (Thermo Fisher Scientific Co, San Jose, CA, USA) with electrospray ionization (ESI) mode was used for detection of gallic acid, p-coumaric acid and quercetin with spray voltage at 3500 V, collision gas at 1.5 arbitrary units, sweep gas flow rate at two arbitrary units, sheath gas flow rate at 38 arbitrary units, auxiliary gas flow rate at 12 arbitrary units, ion transfer tube temperature at 329 °C and vaporizer temperature at 279 °C. Quantitation was carried out using the linear regression equations of the calibration curves of gallic acid, p-coumaric acid and quercetin standards prepared separately by plotting six concentrations of each standard (1, 5, 10, 25, 50 and 100 μg/mL) against its corresponding peak area (quantitative ion intensity).

### 2.8. Preparation of Black Garlic Nanoemulsion and Characteristic Determination

Black garlic nanoemulsion was prepared from 70% EtOH extract as it contains the highest level of total phenolic acids and total flavonoids. A portion (1.25 mL) of concentrated black garlic extract containing phenolic acids and flavonoids at 12,000 μg/mL was evaporated to dryness under nitrogen and 0.12 g grapeseed oil (1.2%) was added and then stirred, followed by adding 0.8 g Tween 80 (8%) and 0.2 g glycerol (2%) for complete mixing. Then 8.88 g deionized water (88.8%) was added, thoroughly mixed, and sonicated for 1 h to obtain a yellow-brown black garlic nanoemulsion with a transparent appearance.

The particle size and distribution of this black garlic nanoemulsion were analyzed by collecting a portion (30 μL) and diluting 100 times with 25 mM dihydrogen potassium phosphate buffer solution (pH 5.3–5.5). Then this solution was filtered through a 0.45-μm membrane filter and poured into a polystyrene colorimetric tube for determination of particle size and distribution by a dynamic light scattering analyzer (DLS) (90 plus model, Brookhaven Instruments Corp, Holtsville, NY, USA).

The zeta potential of this black garlic nanoemulsion was analyzed by collecting a portion (10 μL) and then diluting 120 times with deionized water for determination at 25 °C by a zeta potential analyzer (model SZ100, HORIBA Ltd., Kyoto, Japan).

Also, the particle size and shape of this black garlic nanoemulsion were analyzed by diluting 200 times with deionized water and a portion (20 μL) was collected and dropped onto a carbon coated copper grid for standing for 30 s. Then the excess sample was removed with a filter paper for negative staining with 20 μL phosphotungstic acid (2%) for 60 s, followed by removing excess sample again with a filter paper and drying overnight in an oven for determination by a transmission electron microscope (TEM, model JEM 2100F, JOEL, Tokyo, Japan).

### 2.9. Determination of Encapsulation Efficiency

A portion (100 μL) of the black garlic nanoemulsion was collected, diluted 10 times with 25 mM dihydrogen potassium phosphate buffer solution (pH 5.3–5.5) and poured into a centrifuge tube containing a dialysis membrane (3 kDa) for centrifugation at 12,000 rpm for 20 min (25 °C). Then the lower layer was collected for determination of free (unencapsulated) phenolic acids and flavonoids (gallic acid + p-coumaric acid + quercetin) by using the following formula:

$$\mathrm{Encapsulation}\;\mathrm{efficiency}\;(\%)=\frac{\mathrm{total}\;\mathrm{amount}\;\mathrm{of}\;\mathrm{phenolic}\;\mathrm{acids}\;\mathrm{and}\;\mathrm{flavonoids}-\mathrm{amount}\;\mathrm{of}\;\mathrm{free}\;\mathrm{phenolic}\;\mathrm{acids}\;\mathrm{and}\;\mathrm{flavonoids}}{\mathrm{total}\;\mathrm{amount}\;\mathrm{of}\;\mathrm{phenolic}\;\mathrm{acids}\;\mathrm{and}\;\mathrm{flavonoids}}\times 100$$

### 2.10. Stability Study

The black garlic nanoemulsion was stored at 4 °C for three months, during which a portion of the sample was collected every seven days for determination of particle size, distribution and zeta potential. Similarly, a sample (200 μL) of black garlic nanoemulsion was heated in a water bath for 0.5, 1, 1.5 and 2 h separately with temperatures controlled at 40, 60, 80 and 100 °C.

### 2.11. Animal Study

A total of 42 6-week-old Wistar male rats were purchased from Taiwan BioLASCO Co (Taipei, Taiwan), and these animals were housed in individual ventilation cages with the temperature at 21 ± 2 °C and relative humidity at 55 ± 10% for 12 h under light in Fu Jen University Animal Center. This animal experiment was approved by Fu Jen University animal subjects review committee, while the methods involving animal experiments were performed based on the approved guidelines [18]. All the rats were fed with a laboratory rodent diet (LabDiet Co, St Louis, MO, USA) and water ad libitum. The body weight and water intake of each mouse were measured every week for a total of six weeks. After the rats were acclimatized for two weeks, the eight-week-old rats with an average weight of about 256 g were ready for experiments. Then a total of 42 rats were divided into seven groups with 6 each for administration of the followings for 14 weeks: (1) C (control group), sterilized water (0.3 mL) with subcutaneous injection twice a week for 14 weeks; (2) D (induction group), deoxycorticosterone acetate (DOCA)-salt with subcutaneous injection at 25 mg/kg BW (0.3 mL) twice a week for 14 weeks, while sterilized water (2 mL) containing sodium chloride (1%) and potassium chloride (0.2%) was provided for 14 weeks for induction of hypertension in rats; (3) PC (positive control group), DOCA-salt with subcutaneous injection at 25 mg/kg BW (0.3 mL) twice a week for 14 weeks and tube feeding of lisinopril (hypotensive drug) at 15 mg/kg BW once every day for the last seven weeks; (4) HE (high-dose extract group), DOCA-salt with subcutaneous injection at 25 mg/kg BW (0.3 mL) twice a week for 14 weeks and tube feeding of black garlic extract at 100 mg/kg BW (6 mg of total phenolic acids) once every day for the last seven weeks; (5) HN (high-dose nanoemulsion group), DOCA-salt with subcutaneous injection at 25 mg/kg BW twice (0.3 mL) a week for 14 weeks and tube feeding of black garlic nanoemulsion at 100 mg/kg BW (6 mg of total phenolic acids) once every day for the last seven weeks; (6) LE (low-dose extract group), DOCA-salt with subcutaneous injection at 25 mg/kg BW (0.3 mL) twice a week for 14 weeks and tube feeding of black garlic extract at 50 mg/kg BW (3 mg of total phenolic acids) once every day for the last seven weeks; (7) LN (low-dose nanoemulsion group), DOCA-salt with subcutaneous injection at 25 mg/kg BW (0.3 mL) twice a week for 14 weeks and tube feeding of black garlic nanoemulsion at 50 mg/kg BW (3 mg of total phenolic acids) once every day for the last seven weeks. The blood pressure of each rat was measured once every two weeks. Like D group, all the groups, including PC, HE, HN, LE, and LN, also received sterilized water (2 mL) containing sodium chloride (1%) and potassium chloride (0.2%) for 14 weeks.

### 2.12. Morris Water Maze Test

Two methods based on Morris [19] and Sun et al. [20] were used for the evaluation of memory and learning ability of rats. This test started at the 13th week after feeding and was conducted for seven days with the first three for reference memory test (long-term memory), the fourth day for spatial exploration and the last three for working memory test (short-term memory). The Morris water maze is composed of a round swimming pool with a diameter of 160 cm and height of 60 cm, as well as an escape platform with diameter of 10 cm and a height of 29 cm. Prior to test, water (25 °C) was added to a depth of 30 cm and the platform was below the water surface. The swimming pool was divided into four quadrants (I, II, III and IV) with five different entry points. According to the test difference, the escape platform was placed at the central point of a specific quadrant, while the different shapes of marks (square, triangle, round and cross) were on the swimming pool wall for distinguishing orientation by rats. In addition, a camera was used to record the swimming path of rats.

The reference memory test was carried out from day 84 to day 86. The escape platform was fixed at the central point of the 1st quadrant and rats could enter into water from five entry points randomly. Rats were trained five times with 90 s each every day. If rats could find the escape platform within 90 s, the rats must rest on the platform for 30 s, back to cage for another 30 s before starting the next test. Conversely, if rats could not find the escape platform within 90 s, they must be guided to the escape platform for rest for 30 s, back to cage for another 30 s before starting the next test. This test lasted three days, during which the swimming path and time length of the rats were recorded.

On day 87, the spatial probe test was performed by removing the escape platform from the swimming pool, while rats faced the pool wall and entered into the water from the quadrant IV for swimming for 90 s. The time length spent in the quadrant I and path of full swimming of rats were recorded.

On day 88 to day 90, the working memory test was conducted by placing the escape platform on the central point of different quadrants (II, III and IV), and the rats could enter into water from five entry points randomly. Rats were trained five times with 90 s each every day. If rats could find the escape platform within 90 s, the rats must rest on the platform for 15 s, back to the cage for another 60 s before starting the next test. However, if rats could not find the escape platform within 90 s, they must be guided to the escape platform for rest for 15 s, back to cage for another 60 s before starting the next test. This test lasted three days, during which the swimming path and the time length of rats were recorded.

After the feeding period ended, rats were suffocated with carbon dioxide and then sacrificed. Blood was collected from the celiac vein by a needle, then five mL was collected into a tube without anticoagulant and stood still for 20 min, followed by centrifuging at 3200 *g* for 10 min (4 °C) and collecting the supernatant (serum) for storage at −80 °C. Meanwhile, 5-mL blood was collected into a tube containing the anticoagulant tri-potassium ethylenediaminetetraacetic acid (K3 EDTA) and stood still for 20 min, followed by centrifuging and collecting the upper layer (plasma) for storage at −80 °C. After blood collection, various organs including the heart, liver, kidney, lung and brain were collected, weighed, and stored at −80 °C for further analysis. For the brain organ, the hippocampus part was collected and then 0.1 g mixed with 1 mL of 25 mM phosphoric acid buffer solution (pH 7.4). After homogenization, this solution was centrifuged at 12,000 *g* for 30 min (4 °C) and then the supernatant was collected for storage at −80 °C for further analysis.

### 2.13. Biochemical Analysis of Blood

Both angiotensin II and bradykinin were analyzed respectively using a commercial ELISA kit EKE-002-12 and EK-009-01 (Phoenix Pharmaceuticals Inc, Burlingame, CA, USA). Aldosterone was also analyzed using a commercial radioimmunoassay kit (DSL-8600, Beckman Coulter, Brea, CA, USA). Nitric oxide (NO) was analyzed using a commercial kit (780001, Cayman Chemical Co, Ann Arbor, MI, USA).

### 2.14. Determination of Oxidative Index in Brain Tissue

A method based on Hodges et al. [21] was used to determine malondialdehyde (MDA) content in brain tissue. A 100-μL brain sample or seven different concentrations (5,10, 20, 25, 50, 75 and 100 μM of the standard 1,1,3,3-tetramethoxypropane (TMP) were mixed separately with 200 μL 10% trichloroacetic acid (TCA), after which each mixture was reacted on ice for 15 min to precipitate protein, followed by centrifuging at 3200 *g* for 15 min (4 °C), collecting 200 μL supernatant, adding 200 μL of 0.67% thiobarbituric acid (TBA), reacting in a water bath (100 °C) for 10 min and measuring absorbance at 535 nm for MDA quantitation based on the standard curve.

The superoxide dismutase (SOD) activity was determined using a commercial kit (RANSOD-SD125, Randox Laboratories Ltd., Antrim, UK). The catalase activity was determined using a commercial kit (707002, Cayman Chemical Co, Ann Arbor, MI, USA). The glutathione content was determined using a commercial kit (703002, Cayman Chemical Co, Ann Arbor, MI, USA). The glutathione peroxidase (GSH-Px) activity was determined using a commercial kit (703102, Cayman Chemical Co, Ann Arbor, MI, USA). The acetylcholinesterase (AChE) activity was determined using a commercial kit (ab138871, Abcam Plc, MA, USA).

### 2.15. Determination of Inflammation Index in Brain Tissue

The content of tumor necrosis factor (TNF-α) was determined using a commercial ELISA kit (438207, Biolegend Inc, San Diego, CA, USA). The content of interleukin-1β (IL-1β) was determined using a commercial ELISA kit (900-K91, Pepro Tech Inc, Rocky Hill, NJ, USA). The content of interleukin-6 (IL-6) was determined using a commercial ELISA kit (437107, Biolegend Inc, San Diego, CA, USA).

### 2.16. Statistical Analysis

All the data were subjected to analysis by using Statistical Analysis System [22]. In addition, the variance analysis was conducted by ANOVA, and Duncan’s multiple range test was used for a significant difference in mean comparison (*p* < 0.05).

## 3. Results and Discussion

### 3.1. Analysis of Organosulfur Compounds in Raw and Black Garlic by GC-MS

**Figure 1:**
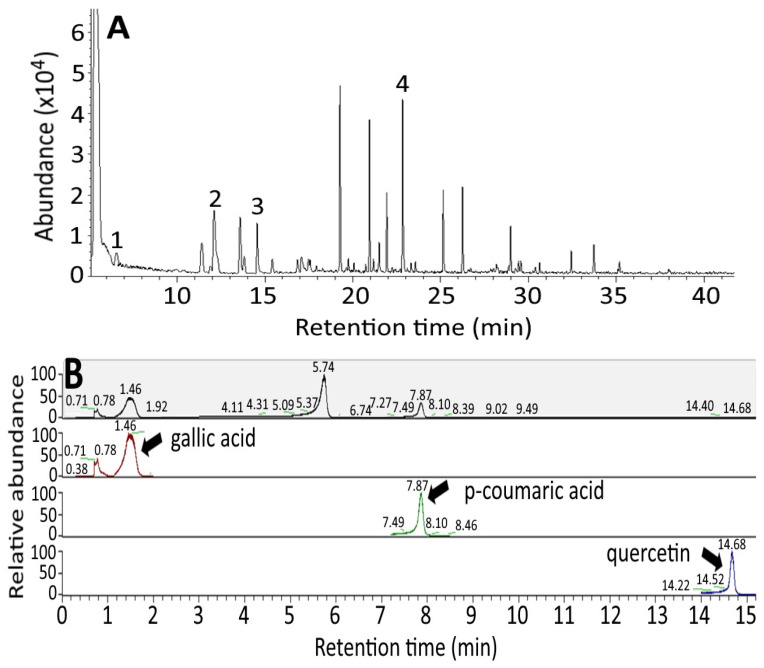

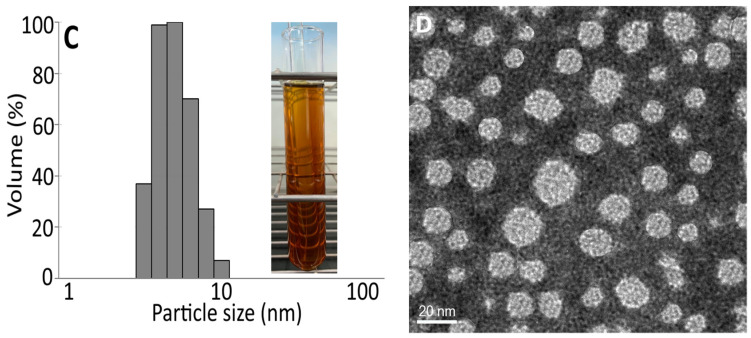
GC-MS chromatogram of black garlic extract using a DB-5MS column (**A**) and UPLC-MS/MS chromatograms of phenolic acid and flavonoids in black garlic with total ion chromatogram shown on the top and the other three being SRM chromatograms (**B**) as well as the particle size distribution of black garlic nanoemulsion by a dynamic light scattering method along with an inset showing its appearance (**C**) and transmission electron microscopic image (**D**). Peaks: 1, diallyl sulfide (DAS); 2, isopropyl disulfide (internal standard); 3, diallyl disulfide (DADS); 4, diallyl trisulfide (DATS).

Figure 1A shows the GC-MS chromatogram of organosulfur compounds in black garlic. Based on the identification criteria shown in the method section, three organosulfur compounds, including diallyl sulfide, diallyl disulfide and diallyl trisulfide were identified in black garlic, with retention time being 6.62, 14.57 and 22.83 min, respectively. Isopropyl disulfide with a retention time at 12.13 min was used as an internal standard to quantify the three organosulfur compounds, with the contents of diallyl sulfide, diallyl disulfide and diallyl trisulfide in black garlic being 87.8, 203.9 and 282.6 μg/g, respectively, based on triplicate determinations (Table 1A). However, in raw garlic, the levels of diallyl sulfide, diallyl disulfide and diallyl trisulfide were 0.93, 2.51 and 3.49 μg/g, respectively. This outcome implied that the level of organosulfur compounds in black garlic was much higher than in raw garlic (Table 1A). The regression equations of the standard calibration curves for diallyl sulfide, diallyl disulfide and diallyl trisulfide were y = 1.4786x − 0.799, y = 0.4217x − 0.1456 and y = 0.6931x − 0.2015, respectively, with R^2^ being all higher than 0.98. For the intra-day variability determination, the RSD (%) for diallyl sulfide, diallyl disulfide and diallyl trisulfide were 3.01, 2.73 and 3.75, respectively, while for the inter-day variability, the RSD (%) were 2.72, 4.77 and 6.67, respectively. All the precision data meets the regulation issued by Taiwan Food and Drug Administration (TFDA) [23], stating that the RSD should be less than 10% when the analyte concentration ≥1 ppm for both the intra-day and the inter-day variability.

The LOD were 0.1, 0.2 and 0.04 μg/mL for diallyl sulfide, diallyl disulfide and diallyl trisulfide, respectively, while the LOQ were 0.4, 0.6 and 0.15 μg/mL. For the accuracy study, the recoveries for diallyl sulfide, diallyl disulfide and diallyl trisulfide were 89.7, 93.5% and 91.1%, respectively. The recovery data also meets the regulation issued by TFDA [23], stating that the recovery should be from 85–110% when the analyte concentration ≥100 ppm. All the data revealed that the method employed in this study for the determination of organosulfur compounds in black garlic possessed high precision and accuracy. In several previous studies, Locatelli et al. [24] reported that diallyl trisulfide was present in the highest amount in home-cooked garlic, followed by diallyl disulfide and diallyl sulfide. However, Tocmo et al. [25] reported that during cooking, allicin could be converted to diallyl disulfide and diallyl trisulfide, both of which may undergo volatilization following prolonged cooking. Zhang et al. [26] further pointed out that the allicin level could decrease during black garlic aging, accompanied by the formation of diallyl disulfide and diallyl trisulfide at high temperature because of allicin instability. Thus, in our study the presence of a high level of diallyl trisulfide, diallyl disulfide and diallyl sulfide in black garlic should be due to the conversion of allicin during aging.

### 3.2. Analysis of Phenolic Acids and Flavonoids in Raw and Black Garlic

Table 1B shows the effect of different ethanol proportion on the contents of total phenolic acids and total flavonoids in raw and black garlic. With 70% ethanol as the extraction solvent, the highest level of total phenolic acids (6.75 mg/g) expressed as gallic acid equivalent (GAE) and total flavonoids (1.28 mg/g) expressed as quercetin equivalent (QE) was shown in black garlic, followed by 50% ethanol and 95% ethanol. A similar trend was shown for raw garlic. In several previous studies, Purev et al. [27] also showed with 70% ethanol as the extraction solvent, the highest yield of total phenolics was obtained in black garlic. In another study, Thach and Thuy [28] also reported that black garlic contained total phenolics and total flavonoids at 7.94 mg GAE/g and 3.37 mg QE/g, respectively. However, a lower level of total phenolics (0.98 mg GAE/g) and total flavonoids (0.87 mg QE/g) in black garlic was reported by Kim, Kang and Gweon [8]. This difference may be accounted for by the difference in aging temperature and relative humidity, time length of aging, garlic variety and solvent variety. By comparison, the levels of total phenolic acids and total flavonoids were much higher in black garlic than in raw garlic (Table 1B), probably caused by the decomposition of the complex of phenolics–protein or phenolics–carbohydrate for the release of free phenolic acids. In addition, the polyphenol compounds may be produced from non-enzymatic browning reaction during aging [7,8].

Figure 1B shows the UPLC-MS/MS chromatograms of phenolic acids and flavonoids in black garlic with total ion chromatogram on the top and selective reaction monitoring (SRM) mode for the other three chromatograms. Of the various phenolic acids and flavonoids, gallic acid, p-coumaric acid and quercetin were identified and quantified with a level of 19.19, 27.03 and 22.77 μg/g, respectively. A similar content of gallic acid (18.65 μg/g), p-coumaric acid (29.51 μg/g) and quercetin (7.31 μg/g) in black garlic was also reported by Kim, Kang and Gweon [8] However, Martínez-Casas, Lage-Yusty and López-Hernández [6] reported a low level of p-coumaric acid (7.5 μg/g) in black garlic, probably caused by the difference in aging condition of the garlic and garlic variety. Compared to black garlic, a much lower level of gallic acid, p-coumaric acid and quercetin was present in raw garlic, which equal to 3.27, 2.25, and 2.06 μg/g, respectively. Thus, black garlic nanoemulsion was prepared for subsequent experiments.

### 3.3. Characteristic of Black Garlic Nanoemulsion

Figure 1C shows the particle size distribution of black garlic nanoemulsion along with an inset depicting its deep-orange and transparent appearance with an average particle size of 10.8 nm, polydispersity index (PDI) of 0.135 and zeta-potential of −60.7 mV (Figure 1C and Table S1). This outcome implied that a narrow distribution of nanoparticles and a high stability of this black garlic nanoemulsion was attained as it was reported that with the PDI value from 0.1 to 0.25, the particle size distribution could be even and narrow in a nanosystem [29]. In addition, with the zeta potential >30 mV or <−30 mV, high stability can be obtained for a nanosystem [29]. Compared to some other nanoemulsions prepared by Mahdi et al. [30] and Baccarin and Lemos-Senna [31], our study showed a much lower PDI and zeta potential, demonstrating a high stability of this black garlic nanoemulsion.

The encapsulation efficiency of the black garlic nanoemulsion was calculated to be 83.2% based on the total phenolic compounds containing gallic acid, p-coumaric acid and quercetin. The content of organosulfur compounds in black garlic nanoemulsion was not used for the determination of encapsulation efficiency mainly because of its volatility, which may undergo loss during the nanoemulsion preparation. This outcome is similar to a study by Bazana et al. [32], preparing a Physalis peruviana calyx nanoemulsion composed of middle-chain triglyceride, Span 80, Tween 80, and deionized water, and reporting an encapsulation efficiency of 84.1%.

Figure 1D shows a TEM image of black garlic nanoemulsion, and an average particle size of 14 nm with a round shape was obtained, which is similar to that (10.8 nm) measured by DLS. This size is much smaller than the study by Mahdi, Noor, Sakeena, Abdullah, Abdulkarim and Sattar [30], preparing total phenolics nanoemulsion from *Phyllanthus urinaria* and reporting an average particle size of 30.74 nm.

Table S1 shows particle size, PDI and zeta potential of black garlic nanoemulsion over a 90-day storage period at 4 °C. Only a minor change in particle size, PDI and zeta potential was shown during storage, implying a high stability of the black garlic nanoemulsion prepared in our study. Similarly, this black garlic nanoemulsion also showed a high stability during heating at 40, 60, 80 and 100 °C for 0.5, 1 and 2 h, as evident by a slight change in particle size and PDI (Table S2). However, the zeta potential increased to >−30 mV when the temperature was ≥80 °C and heating time ≥1 h, indicating that the heating condition should be controlled carefully to obtain the highest stability of this black garlic nanoemulsion.

### 3.4. Animal Study

Table S3 shows the effect of the administration of black garlic extract and nanoemulsion on body weight in DOCA-salt-induced hypertension and its associated mild cognitive impairment in rats. A time-dependent increase in the body weight of rats was observed over a 13-week feeding period for all the groups. Compared to C (control) group, the body weight in rats for D, PC, HE, HN, LE and LN groups were significantly lower (*p* < 0.05) by 11.5%, 11.9%, 11.9%, 9.1%, 8.2% and 12.1%, respectively. However, there was no significant difference (*p* > 0.05) in body weight of rats between D and the other five groups (PC, HE, HN, LE and LN). The daily water intake showed an inconsistent change over a 13-week feeding period for all the groups (Table S4). Compared to C group on week 13, the water intake by rats for D, PC, HE, HN, LE and LN groups were significantly higher (*p* < 0.05) by 3.23-, 3.59-, 3.45-, 3.64-, 3.43- and 3.48-fold, respectively. However, there was no significant difference (*p* > 0.05) in water intake between D and the other five groups (PC, HE, HN, LE and LN). This result is expected as both sodium chloride and potassium chloride were added to drinking water and hence the water demand for D and the other five groups should be enhanced greatly. In addition, DOCA, a kind of mineral corticoid, can not only combine with its receptor to enhance sodium retention and potassium excretion for maintenance of body balance but also stimulate salt appetite in rats to increase saltwater intake [33,34].

Table 2 shows the effect of the administration of black garlic extract and nanoemulsion on systolic blood pressure in DOCA-salt-induced hypertension and its associated mild cognitive impairment in rats. Initially there was no significant difference (*p* > 0.05) in systolic blood pressure in rats between C and the other 6 groups. However, this difference became more pronounced after the feeding time reached 4 weeks. Following 8-week administration, the systolic blood pressure in rats for D, PC, HE, HN, LE and LN groups were significantly higher (*p* < 0.05) than C group by 49.9, 41.0, 44.5, 42.8, 44.5 and 42.7 mmHg, respectively, indicating that a high-blood-pressure animal model was successfully established on week 8. Following administration for another 4 weeks (week 12), the systolic blood pressure in rats could be reduced by 29.0, 23.4, 24.0, 28.4 and 21.1 mmHg for PC, HE, HN, LE and LN groups, respectively, compared to D group. This finding clearly revealed that the hypotensive drug lisinopril and both high-dose black garlic extract and nanoemulsion were the most effective in reducing blood pressure in DOCA-salt-induced rats. Thus, an optimal dose of black garlic extract and nanoemulsion is required to reduce systolic blood pressure in DOCA-salt-induced rats.

In the literature, most studies deal with the effect of raw garlic extract, not black garlic, on reduction of blood pressure in rats. For instance, following administration of garlic extract at 50 mg/kg BW by tube feeding for seven days, the systolic blood pressure in rats was significantly reduced (*p* < 0.05) by 29 mmHg compared to the induction group. Similar outcomes were reported by Asdaq and Inamdar [35] and Sharifi et al. [36], showing a reduction of systolic blood pressure in rats by 53.9 and 30 mmHg, respectively, following tube feeding of garlic extract at 250 mg/kg BW for three weeks for the former and at 50 mg/kg BW for four weeks for the latter. Apparently, the efficiency of blood pressure reduction in rats can be dependent upon administration dose, feeding period and garlic extract composition. Moreover, the reduction of systolic blood pressure in rats can be due to maintenance of blood vessel elasticity and erythrocyte deformability for improvement in blood circulation. Nevertheless, Harauma and Moriguchi [37] pointed out that a high dose of garlic extract may cause side effects such as erythrocyte reduction in stomach and papilloma. In addition, a high dose of garlic extract (500 mg/kg BW) was shown to be less effective in reducing blood pressure than that of a medium dose (250 mg/kg BW), probably caused by incomplete conversion of allicin to active organosulfur compounds [35]. Thus, compared to raw garlic, black garlic should be less irritating to the stomach as allicin can be degraded during aging [14].

**Figure 2:**
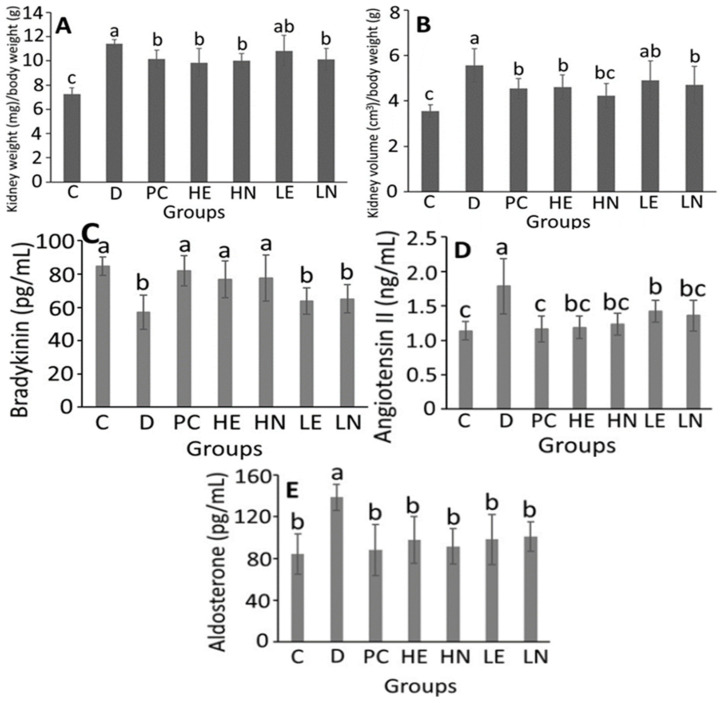
Effect of the administration of black garlic extract and nanoemulsion onkidney weight (**A**), kidney volume (**B**), bradykinin (**C**), angiotensin II (**D**) and aldosterone (**E**) in DOCA-salt-induced hypertension and its associated mild cognitive impairment in rats. Data are presented as means ± standard deviation (*n* = 6); C, control; D, DOCA-salt at a dose 25 mg/kg BW; PC, DOCA-salt with the administration of lisinopril at a dose 15 mg/kg BW; HE, DOCA-salt with the administration of black garlic extract at a dose of 100 mg/kg BW; HN, DOCA-salt with the administration of black garlic nanoemulsion at a dose of 100 mg/kg BW; LE, DOCA-salt with the administration of black garlic extract at a dose of 50 mg/kg BW; LN, DOCA-salt with the administration of black garlic nanoemulsion at a dose of 50 mg/kg BW. Values with different small letters (a–c) are significantly different (*p* < 0.05).

Figure 2A,B show the effect of the administration of black garlic extract and nanoemulsion respectively on kidney weight and volume in DOCA-salt-induced hypertension and its associated mild cognitive impairment in rats. It has been well documented that in DOCA-salt-induced rats, the kidney function can be injured seriously through glomerulosclerosis, tubular fibrosis and tubular dilation [38]. Compared to C group, the ratio of kidney weight (mg)/body weight (g) in rats for D, PC, HE, HN, LE and LN groups were significantly higher (*p* < 0.05) by 1.58-, 1.40- 1.35-, 1.36-, 1.49- and 1.40-fold, respectively. However, no significant difference (*p* > 0.05) was observed among the PC, HE, HN, LE and LN groups. A similar tendency was shown for the ratio of kidney volume (cm^3^)/body weight (g). By comparison, PC, HE, HN, and LN groups showed a significantly lower (*p* < 0.05) ratio of kidney weight (mg)/body weight (g) than D group by 11.1, 13.7, 12.4 and 11.5%, respectively. But for the ratio of kidney volume (cm^3^)/body weight (g), PC, HE, HN, and LN groups were significantly lower (*p* < 0.05) than D group by 18.4, 17.3, 23.9 and 15.5%, respectively, demonstrating that all the PC, HE, HN, and LN groups could be protective against DOCA-salt-induced kidney impairment in rats. This finding is similar to several previous studies, showing the ratio of kidney weight/body weight in DOCA-salt-induced rats to be higher than C group by 1.76-fold [38], 2.96-fold [39]. and 1.98-fold [40], following administration of hemin, α-lipoic acid and L-carnitine, respectively.

The concentration changes of bradykinin, angiotensin II and aldosterone in DOCA-salt-induced hypertension and its associated mild cognitive impairment in rats as affected by black garlic extract and nanoemulsion is shown in Figure 2C–E. Compared to C group, the plasma bradykinin concentration in D group was significantly reduced (*p* < 0.05) by 27.75 pg/mL (Figure 2C). Of the various groups, only PC, HE and HN could significantly increase (*p* < 0.05) the plasma bradykinin concentration by 25.00, 19.86, and 20.67 pg/mL, respectively, when compared to D group, while both LE and LN groups showed no significant difference (*p* > 0.05).

In Figure 2D, the angiotensin II (Ang II) concentration in plasma of DOCA-salt-induced hypertension rats was significantly higher (*p* < 0.05) than C group by 0.64 ng/mL. However, compared to D group, the Ang II concentration could be significantly reduced (*p* < 0.05) by 0.62, 0.59, 0.55, 0.35 and 0.42 ng/mL for PC, HE, HN, LE and LN groups, respectively. Interestingly, there was no significant difference (*p* > 0.05) in Ang II concentration among PC, HE, HN, LE and LN groups.

In Figure 2E, the aldosterone concentration in plasma of DOCA-salt-induced hypertension rats was significantly higher (*p* < 0.05) than C group by 54.25 pg/mL. However, compared to D group, the aldosterone concentration could be significantly reduced (*p* < 0.05) by 50.80, 40.94, 47.15, 40.43 and 37.56 pg/mL for PC, HE, HN, LE and LN groups, respectively, implying that lisinopril was the most effective in reducing aldosterone concentration in rat plasma, followed by high-dose black garlic nanoemulsion. Accordingly, lisinopril, a frequently used anti-hypertension drug, is also an Ang I converting enzyme inhibitor. It has been established that ACE I can combine with ACE to inhibit ACE activity, thereby reducing the formation of Ang II and degradation of bradykinin, and leading to vasodilation and a decrease of blood pressure [41]. Furthermore, the renin-angiotensin-aldosterone system (RAAS) can catalyze the hydrolysis of angiotensinogen from liver into non-active angiotensin I (Ang I), which in turn can be converted to active Ang II through angiotensin-converting enzyme (ACE) for subsequent contraction of vascular smooth muscle and stimulation of adrenal cortex to secrete aldosterone, leading to enhancement of sodium ion absorption by kidney and elevation of blood pressure [42]. In addition, bradykinin can be converted to non-active peptide fragments in the presence of ACE, thereby inhibiting vasodilation [41]. In several previous studies using the same animal model, the elevation of bradykinin concentration and decrease of aldosterone concentration in rat plasma by a fermented product NTU101 [43], as well as reduction of Ang II concentration in rat plasma by apelin, an active polypeptide [44], were demonstrated.

Collectively, both black garlic extract and nanoemulsion prepared in our study could reduce blood pressure in rats through an increase of bradykinin concentration and decrease of both Ang II and aldosterone concentrations in rat plasma with lisinopril, high dose of both black garlic extract and nanoemulsion being the most efficient.

### 3.5. Morris Water Maze Test

Table 3 shows the effect of the administration of black garlic extract and nanoemulsion on reference memory task in Morris water maze test in DOCA-salt-induced hypertension and its associated mild cognitive impairment in rats. Compared to D group, the swimming distance for rats to reach the escape platform could be significantly reduced (*p* < 0.05) by 273.07, 275.38, 297.29, 250.11 and 253.74 cm for PC, HE, HN, LE and LN groups, respectively. However, no significant difference (*p* > 0.05) in swimming distance was observed among PC, HE, HN, LE and LN groups. Similarly, compared to D group, the escape latency for PC, HE, HN, LE and LN groups was significantly reduced (*p* < 0.05) by 14.21, 14.59, 14.82, 13.06 and 13.20 s, respectively. In addition, no significant difference (*p* > 0.05) in escape latency was shown among PC, HE, HN, LE and LN groups. Likewise, compared to C group, the time length of rats in the target quadrant was significantly reduced (*p* < 0.05) by 16.15 s for D group (Table S5). However, when compared to D group, all the PC, HE, HN, LE and LN groups increased time length of rats in the target quadrant by 15.21, 12.22, 12.46, 5.17 and 7.88 s, respectively (Table S5). Similarly, for average of the other three quadrants, the PC, HE and HN groups could reduce time length of rats by 5.41, 4.18 and 4.27 s, respectively (Table S5 and Figure S1), suggesting that lisinopril and both high-dose black garlic extract and nanoemulsion were more effective in improving long-term memory ability than low-dose black garlic extract and nanoemulsion during the spatial probe test.

Table 3 also shows the effect of the administration of black garlic extract and nanoemulsion on working memory task in Morris water maze in DOCA-salt-induced hypertension and its associated mild cognitive impairment in rats. On day 5, 6 and 7, D group showed a significantly higher (*p* < 0.05) swimming distance and escape latency than C group. However, compared to D group on day 7, all the PC, HE, HN, LE and LN groups could significantly reduce (*p* < 0.05) swimming distance by 273.07, 275.38, 297.29, 250.11 and 253.74 cm, respectively, as well as escape latency by 14.21, 14.59, 14.82, 13.06 and 13.20 s. This outcome further demonstrated that lisinopril and both high-dose black garlic nanoemulsion and extract possessed a more pronounced effect than low-dose black garlic nanoemulsion and extract in improving short memory ability in DOCA-salt-induced hypertension and its associated mild cognitive impairment in rats.

In several previous studies Hermawati et al. [45], reported that a dose (50 mg/kg BW) of black garlic extract could improve space memory ability of monosodium glutamate-induced rats in a Morris maze text. Following intraperitoneal injection of S-allyl cysteine, a major organosulfur compound in black garlic at 30 mg/kg BW to mice for 15 days, the swimming distance and escape latency was reduced by 43% and 32%, respectively, compared to induction group. This outcome indicated that S-allyl cysteine was effective in preventing cognitive impairment in rats [46]. Likewise, compared to induction group, the administration of garlic extract to rats at 250 mg/kg BW could reduce escape latency by 47% and increase time length in the target quadrant by 60% in a Morris maze test [47], demonstrating again that garlic extract was effective in improving cognitive function. Similar finding was reported by Ghasemi et al. [48] showing a rise of the target quadrant time length by 45% in a Morris maze test, as well as an increase of latency time to the dark environment by sevenfold in a passive avoidance test following administration of garlic juice at 100 mg/kg BW to rats.

Taken together, both garlic extract and nanoemulsion were effective in improving learning and memory ability of rats. However, the efficiency may be varied depending on the variety and amount of bioactive compounds in fresh garlic and black garlic. As mentioned before, black garlic contained a higher level of bioactive compounds including diallyl sulfide, diallyl disulfide and diallyl trisulfide, phenolic acids and flavonoids, and thus a more pronounced effect in improving cognitive function ability in rats was observed when compared to many studies dealing with raw garlic extract. However, Farooqui and Farooqui [49] further pointed out that all the bioactive compounds in fresh garlic or black garlic prevent neurodegeneration through the reduction of oxidative pressure and nerve inflammation. Nevertheless, our study further demonstrated that black garlic nanoemulsion was more effective than raw garlic extract in improving cognitive function ability in rats.

### 3.6. Determination of NO Concentration in Plasma, Oxidative and Inflammation Index in Hippocampus in DOCA-Salt Induced Hypertension and Its Associated Mild Cognitive Impairment in Rats

Table 4 shows the effect of the administration of black garlic extract and nanoemulsion on the nitric oxide (NO) concentration in plasma, contents of TNF-α, IL-6 and IL-1β, as well as activities of acetylcholinesterase (AChE), superoxide dismutase (SOD), catalase (CAT), glutathione (GSH), glutathione peroxidase (GSH-Px) and malondialdehyde (MDA) concentrations in hippocampus in DOCA-salt-induced hypertension and its associated mild cognitive impairment in rats. Compared to C group, the NO concentration in D group was reduced by 10.34 μM. Of the various groups, only PC, HE and HN could significantly increase (*p* < 0.05) NO concentration by 10.75, 7.21 and 8.29 μM, respectively, when compared to D group. It has been well established that in DOCA-salt-induced rats, the hydroperoxide substance can be increased in blood vessel to decrease NO concentration through the reaction with NO and peroxynitrite formation, leading to endothelial contraction disorder in blood vessels [50,51]. Moreover, following administration of both high-dose black garlic extract and nanoemulsion, the presence of both phenolics and flavonoid compounds could be effective antioxidants in reducing peroxynitrite formation for elevation of NO concentration and reduction of blood pressure in DOCA-salt-induced rats.

Acetylcholine, a vital neurotransmitter in brain, can be synthesized and released into synapse for reaction with its receptor for regulation of neurotransmitter release and down-stream message transfer. However, when acetylcholine is present in excess, it can be decomposed into choline and acetyl CoA by AChE for recycle and reuse [52]. Compared to C group, the AChE activity in hippocampus of rats for D group was significantly higher (*p* < 0.05) by 24.05 mU/g. However, following administration of PC, HE, HN, LN and LE, the AChE activity in hippocampus of rats was significantly reduced (*p* < 0.05) by 29.55, 26.41, 28.40, 21.91 and 16.10 mU/g, respectively, when compared to D group. Apparently, the presence of AChE in excess can result in deficiency of acetylcholine for subsequent impairment of memory and cognition [53]. A similar trend was observed for MDA, an index of lipid peroxidation, i.e., the MDA level in hippocampus of rats for D group was significantly higher (*p* < 0.05) than C group by 27.77 nmol/g. However, following administration of PC, HE, HN LN and LE, the MDA level was reduced by 25.43, 19.64, 23.71, 4.37 and 7.75 nmol/g, respectively. For SOD activity in hippocampus of rats, D group was significantly lower (*p* < 0.05) than C group by 11.03 U/g. However, following administration of PC, HE, HN, LN and LE, the SOD activity significantly increased (*p* < 0.05) by 12.04, 11.23, 9.74, 4.15 and 6.68 U/g, respectively, when compared to D group. Similar findings were observed for CAT, GSH and GSH-Px. The CAT activity in hippocampus of rats for D group was significantly lower (*p* < 0.05) than C group by 234.22 mU/g. However, following administration of PC, HE, HN, LE and LN, the CAT activity significantly increased (*p* < 0.05) by 176.67, 88.52, 117.92, 39.94 and 72.32 mU/g, respectively, when compared to D group. For the GSH and GSH-Px activity in hippocampus of rats, D group was significantly lower (*p* < 0.05) than C group by 214.6 nmol/g and 1.99 nmol/min/g, respectively. However, compared to D group, a steep rise (*p* < 0.05) of GSH by 178.21, 208.05 and 234.65 nmol/g as well as GSH-Px by 1.66, 1.22 and 1.36 nmol/min/g was respectively shown for PC, HE and HN groups. Comparatively, all the PC, HE and HN groups were the most effective in enhancing activities of antioxidant enzymes including SOD, CAT, GSH and GSH-Px, while minimizing MDA production. Apparently, the enhanced antioxidant activity of black garlic can be associated with phenolic acid, flavonoid and organosulfur compounds, resulting in a higher antioxidant activity than fresh garlic. In a previous study Li and Kim [54] also reported that following administration of black garlic extract at 50 mg/kg BW for 4 weeks by tube feeding, both GSH and GSH-Px levels in hippocampus of rats could increase by 50 and 45%, respectively, while both AChE and MDA levels reduced by 32 and 24%. In addition, Kumar [55] demonstrated that the inhibition of AChE and butyryl cholinesterase (BChE) activities can be attributed to the presence of major bioactive compounds in garlic such as allicin. Nevertheless, both phenolic acids and flavonoids in black garlic nanoemulsion should also play a vital role in inhibiting AChE and BChE activities. Likewise, following tube feeding of garlic extract to rats at 250 mg/kg BW for 9 weeks, the activities of SOD, GSH-Px and CAT rose by 102%, 117% and 34%, respectively, while the MDA level reduced by 58%. Obviously, the difference in antioxidant activity of fresh garlic or black garlic can also be caused by difference in animal model, dose, sample variety, feeding period and method of determining oxidative index. Collectively, the drug lisinopril as well as both high-dose black garlic extract and nanoemulsion were the most effective in enhancing SOD, CAT, GSH and GSH-Px activities while minimizing both AChE and MDA levels.

For the inflammation index, compared to C group, the levels of TNF-α, IL-6 and IL-1β were significantly higher (*p* < 0.05) by 283.22, 794.00 and 1367.63 pg/g for D group, respectively. However, following administration of PC, HE, HN, LE and LN, the TNF-α level was reduced by 216.31, 191.87, 203.18, 103.40 and 116.12 pg/g, respectively, when compared to D group. Likewise, compared to D group, the levels of IL-6 were reduced by 689.89, 457.61, 637.92, 379.58 and 268.08 pg/g for PC, HE, HN, LE and LN groups, respectively, as well as 1233.15, 910.44, 820.68, 268.67 and 472.18 pg/g for PC, HE, HN, LE and LN groups for the IL-1β level. By comparison, the PC, HE and HN groups were the most effective in reducing TNF-α, IL-6 and IL-1β levels. Likewise, following administration of fresh garlic or black garlic extracts by tube feeding at 120 mg/kg BW, the TNF-α level in serum was reduced by 56 and 52%, respectively, as well as IL-6 level by 10% and 15%, when compared to the induction group [10]. In addition, after tube feeding of raw garlic extract at 250 mg/kg BW, the IL-1β level was reduced by 50 %. All these studies suggested that both fresh garlic and black garlic extracts possessed anti-inflammatory effect. Nevertheless, raw garlic extract was shown to possess a higher cell toxicity towards macrophage RAW 264.7 than black garlic extract [10]. Thus, from the safety point of view, black garlic should be more important than fresh garlic for future biomedical applications.

## 4. Conclusions

In conclusion, both black garlic extracts and nanoemulsions can improve DOCA-salt-induced hypertension in rats through an increase of bradykinin concentration and decrease of aldosterone and Ang II concentrations as well as increase of NO production, while the learning and memory ability of rats can be improved through reduction of AChE activity, oxidative stress and inflammation as well as enhancement of antioxidant enzyme activity (Figure S2). Further research is necessary to explore the possibility of future clinical trial for treatment of patients with dementia disease.

## Figures and Tables

**Table 1:** (**A**) Contents (μg/g) of organosulfur compounds in raw and black garlic by GC-MS analysis. (**B**) Effect of different ethanol proportion on the contents of total phenolic acids and total flavonoids in raw and black garlic by UPLC-MS/MS analysis.

(A)						
	Diallyl Sulfide		Diallyl Disulfide		Diallyl Trisulfide	
Raw garlic	0.93 ^c^		2.51 ^b^		13.49 ^a^	
Black garlic	87.8 ^c^		203.9 ^b^		282.6 ^a^	
**(B)**						
	**50% EtOH**		**70% EtOH**		**95% EtOH**	
	**Raw Garlic**	**Black Garlic**	**Raw Garlic**	**Black Garlic**	**Raw Garlic**	**Black Garlic**
Total phenolic acids ^A^	0.590 ± 0.049 ^e^	5.49 ± 0.35 ^b^	0.641 ± 0.023 ^d^	6.75 ± 0.46 ^a^	0.534 ± 0.024 ^f^	1.40 ± 0.05 ^c^
Total flavonoids ^B^	0.044 ± 0.006 ^e^	0.75 ± 0.05 ^b^	0.120 ± 0.010 ^d^	1.28 ± 0.12 ^a^	0.025 ± 0.006 ^f^	0.15 ± 0.01 ^c^

^A^ Data expressed as mg/g of gallic acid equivalent (GAE). ^B^ Data expressed as mg/g quercetin equivalent (QE). Data are presented as mean ± standard deviation of triplicate determinations for total phenolic acids and total flavonoids. Data with different small letters (a–c) in Table 1A and (a–f) in Table 1B are significantly different at *p* < 0.05.

**Table 2:** Effect of the administration of black garlic extract and nanoemulsion on systolic blood pressure in DOCA-salt-induced hypertension and its associated mild cognitive impairment in rats.

Groups	Systolic Blood Pressure (mmHg)						
	Week 0	Week 2	Week 4	Week 6	Week 8	Week 10	Week 12
C	117.2 ± 3.5 ^Ba^	121.4 ± 3.6 ^ABb^	120.3 ± 6.4 ^ABb^	123.2 ± 3.9 ^Ab^	117.0 ± 4.9 ^Bc^	119.2 ± 4.2 ^ABc^	120.2 ± 4.9 ^ABd^
D	118.5 ± 2.9 ^Ea^	126.0 ± 4.0 ^Dab^	137.5 ± 6.3 ^Ca^	160.0 ± 6.3 ^Ba^	166.9 ± 5.1 ^Aa^	169.5 ± 7.7 ^Aa^	173.4 ± 4.4 ^Aa^
PC	120.5 ± 4.8 ^Da^	127.7 ± 6.6 ^Cab^	138.4 ± 8.2 ^Ba^	158.3 ± 5.7 ^Aa^	158.0 ± 4.6 ^Ab^	152.6 ± 6.9 ^Ab^	144.4 ± 7.3 ^Bc^
HE	119.4 ± 4.5 ^Da^	125.6 ± 6.5 ^Dab^	136.8 ± 9.5 ^Ca^	157.2 ± 9.0 ^ABa^	161.5 ± 7.7 ^Aab^	156.7 ± 6.6 ^ABb^	150.0 ± 7.6 ^Bbc^
HN	118.6 ± 3.6 ^Ea^	129.0 ± 5.8 ^Da^	141.3 ± 4.9 ^Ca^	158.7 ± 7.0 ^Aa^	159.8 ± 8.7 ^Aab^	155.2 ± 6.8 ^Ab^	149.4 ± 8.1 ^Bbc^
LE	116.8 ± 5.1 ^Da^	124.8 ± 8.5 ^Cab^	144.5 ± 6.7 ^Ba^	162.7 ± 6.5 ^Aa^	161.5 ± 6.7 ^Aab^	158.2 ± 8.0 ^Ab^	155.0 ± 8.0 ^Ab^
LN	119.1 ± 6.0 ^Da^	129.5 ± 5.6 ^Ca^	142.1 ± 6.3 ^Ba^	160.2 ± 7.1 ^Aa^	159.7 ± 7.3 ^Aab^	156.3 ± 8.2 ^Ab^	152.3 ± 5.3 ^Abc^

Data are presented as means ± standard deviation (*n* = 6); C, control; D, DOCA-salt at a dose 25 mg/kg BW; PC, DOCA-salt with the administration of lisinopril at a dose 15 mg/kg BW; HE, DOCA-salt with the administration of black garlic extract at a dose of 100 mg/kg BW; HN, DOCA-salt with the administration of black garlic nanoemulsion at a dose of 100 mg/kg BW; LE, DOCA-salt with the administration of black garlic extract at a dose of 50 mg/kg BW; LN, DOCA-salt with the administration of black garlic nanoemulsion at a dose of 50 mg/kg BW. Values with different capital letters (A–E) in the same row and small letters (a–d) in the same column are significantly different (*p* < 0.05).

**Table 3:** Effect of the administration of black garlic extract and nanoemulsion on reference and working memory task in Morris water maze in DOCA-salt-induced hypertension and its associated mild cognitive impairment in rats.

Group	Total Swimming Distance (cm)			Escape Latency (s)		
	Day 1	Day 2	Day 3	Day 1	Day 2	Day 3
Reference memory task						
C	767.73 ± 103.73 ^Aa^	441.75 ± 112.66 ^Bb^	224.02 ± 97.25 ^Cb^	31.87 ± 10.15 ^Aa^	18.92 ± 5.85 ^Bb^	9.92 ± 4.29 ^Bb^
D	995.84 ± 188.73 ^Aa^	713.38 ± 209.50 ^ABa^	541.26 ± 205.86 ^Ba^	50.24 ± 11.48 ^Aa^	39.19 ± 8.04 ^ABa^	28.44 ± 9.08 ^Ba^
PC	842.06 ± 313.78^Aa^	371.96 ± 173.97 ^Bb^	281.97 ± 163.49 ^Bb^	34.33 ± 13.37 ^Aa^	16.67 ± 7.05 ^Bb^	13.61 ± 7.11 ^Bb^
HE	791.77 ± 182.28 ^Aa^	439.65 ± 182.25 ^Bb^	307.71 ± 75.56 ^Bb^	37.13 ± 12.21 ^Aa^	19.89 ± 7.01 ^Bb^	14.57 ± 5.38 ^Bb^
HN	746.88 ± 269.54 ^Aa^	359.68 ± 129.05 ^Bb^	250.27 ± 125.73 ^Bb^	36.25 ± 13.51 ^Aa^	16.28 ± 6.31 ^Bb^	12.21 ± 5.65 ^Bb^
LE	907.87 ± 294.82 ^Aa^	444.94 ± 78.91 ^Bb^	301.83 ± 108.25 ^Bb^	43.02 ± 12.34 ^Aa^	21.01 ± 2.67 ^Bb^	15.44 ± 4.57 ^Bb^
LN	890.78 ± 343.01 ^Aa^	428.43 ± 137.43 ^Bb^	375.43 ± 185.71 ^Bab^	41.31 ± 20.36 ^Aa^	20.11 ± 6.31 ^Bb^	18.64 ± 5.92 ^Bb^
	**Day 5**	**Day 6**	**Day 7**	**Day 5**	**Day 6**	**Day 7**
Working memory task						
C	193.56 ± 114.26 ^A^	156.76 ± 43.02 ^B^	142.78 ± 34.08 ^B^	8.93 ± 6.14 ^A^	6.73 ± 2.06 ^B^	6.94 ± 1.93 ^B^
D	783.07 ± 123.26 ^A^	552.35 ± 220.46 ^B^	439.66 ± 101.02 ^B^	35.91 ± 6.61 ^A^	28.12 ± 7.57 ^A^	21.52 ± 3.12 ^B^
PC	247.31 ± 137.00 ^A^	231.64 ± 89.02 ^A^	166.59 ± 73.52 ^B^	10.49 ± 7.11 ^A^	8.93 ± 3.09 ^B^	7.31 ± 2.53 ^B^
HE	306.80 ± 92.97 ^A^	281.27 ± 81.48 ^A^	164.28 ± 91.38 ^B^	14.43 ± 5.22 ^A^	13.48 ± 4.54 ^A^	6.93 ± 3.21 ^B^
HN	323.90 ± 107.48 ^A^	243.63 ± 85.15 ^B^	142.37 ± 98.54 ^C^	14.87 ± 6.05 ^A^	11.33 ± 4.21 ^A^	6.70 ± 5.61 ^B^
LE	426.64 ± 126.97 ^A^	298.02 ± 78.58 ^B^	189.55 ± 66.06 ^C^	18.23 ± 5.67 ^A^	12.92 ± 1.30 ^B^	8.46 ± 3.01 ^C^
LN	447.39 ± 119.98 ^A^	283.72 ± 94.82 ^BC^	185.92 ± 105.31 ^C^	9.43 ± 6.26 ^A^	7.58 ± 3.13 ^B^	8.32 ± 4.12 ^B^

Data are presented as means ± standard deviation (*n* = 6); C, control; D, DOCA-salt at a dose 25 mg/kg BW; PC, DOCA-salt with the administration of lisinopril at a dose 15 mg/kg BW; HE, DOCA-salt with the administration of black garlic extract at a dose of 100 mg/kg BW; HN, DOCA-salt with the administration of black garlic nanoemulsion at a dose of 100 mg/kg BW; LE, DOCA-salt with the administration of black garlic extract at a dose of 50 mg/kg BW; LN, DOCA-salt with the administration of black garlic nanoemulsion at a dose of 50 mg/kg BW. Values with different capital letters (A–C) in the same row for total swimming distance or escape latency and small letters (a,b) in the same column for total swimming distance or escape latency are significantly different (*p* < 0.05).

**Table 4:** Effect of the administration of black garlic extract and nanoemulsion on nitric oxide concentration in plasma and contents of tumor necrosis factor-α (TNF-α), interleukin-6 (IL-6) and interleukin-1β (IL-1β) as well as activities of acetylcholinesterase (AChE), superoxide dismutase (SOD), catalase (CAT), glutathione (GSH), glutathione peroxidase (GSH-Px) and malondialdehyde (MDA) concentrations in hippocampus in DOCA-salt-induced hypertension and its associated mild cognitive impairment in rats.

Groups	Nitrate + Nitrite (μM)		TNF-α (pg/g Tissue)		IL-6 (pg/g Tissue)		IL-1β (pg/g Tissue)	
C	23.28 ± 5.95 ^A^		310.53 ± 79.33 ^C^		1890.75 ± 84.36 ^D^		1465.38 ± 118.46 ^D^	
D	12.94 ± 3.54 ^C^		593.75 ± 121.77 ^A^		2684.75 ± 281.72 ^A^		2833.01 ± 208.09 ^A^	
PC	23.69 ± 5.07 ^A^		377.44 ± 54.96 ^BC^		1994.86 ± 215.67 ^CD^		1599.86 ± 166.94 ^D^	
HE	20.15 ± 3.81 ^AB^		401.88 ± 94.12 ^BC^		2227.14 ± 241.24 ^BC^		1922.57 ± 130.81 ^C^	
HN	21.23 ± 5.08 ^AB^		390.57 ± 78.31 ^BC^		2046.83 ± 123.61 ^CD^		2012.33 ± 267.47 ^C^	
LE	15.98 ± 3.87 ^BC^		490.35 ± 107.76 ^AB^		2305.17 ± 194.02 ^B^		2564.34 ± 208.08 ^B^	
LN	15.65 ± 3.94 ^BC^		477.63 ± 97.51 ^B^		2416.67 ± 175.18 ^B^		2360.83 ± 200.97 ^B^	
**Groups**	**AChE** **(mU/g Tissue)**	**SOD** **(U/g Tissue)**		**CAT** **(mU/g Tissue)**	**GSH** **(nmol/g Tissue)**	**GSH-Px** **(nmol/min/g Tissue)**		**MDA** **(nmol/g Tissue)**
C	56.73 ± 14.27 ^B^	44.80 ± 3.15 ^A^		514.93 ± 76.89 ^A^	631.83 ± 64.16 ^A^	3.91 ± 1.06 ^A^		113.84 ± 10.39 ^B^
D	80.78 ± 16.41 ^A^	33.77 ± 7.89 ^B^		280.71 ± 70.29 ^D^	417.23 ± 69.74 ^B^	1.92 ± 0.57 ^D^		141.61 ± 14.77 ^A^
PC	51.23 ± 8.06 ^B^	45.81 ± 6.02 ^A^		457.38 ± 114.79 ^AB^	595.44 ± 68.85 ^A^	3.58 ± 0.71 ^A^		116.18 ± 8.89 ^B^
HE	54.37 ± 9.97 ^B^	45.00 ± 5.95 ^A^		369.23 ± 90.53 ^C^	625.28 ± 96.64 ^A^	3.14 ± 0.70 ^AB^		121.97 ± 8.30 ^B^
HN	52.38 ± 24.09 ^B^	43.51 ± 4.74 ^A^		398.63 ± 73.87 ^C^	651.88 ± 65.66 ^A^	3.28 ± 0.67 ^AB^		117.90 ± 4.88 ^B^
LE	64.68 ± 12.40 ^B^	37.92 ± 7.31 ^AB^		320.65 ± 61.41 ^CD^	427.19 ± 82.71 ^B^	2.25 ± 0.78 ^C^		137.24 ± 7.00 ^A^
LN	58.87 ± 11.14 ^B^	40.45 ± 9.29 ^AB^		353.03 ± 99.85 ^C^	463.07 ± 47.70 ^B^	2.55 ± 0.76 ^C^		133.86 ± 11.45 ^A^

Data are presented as means ± standard deviation (*n* = 6); C, control; D, DOCA-salt at a dose 25 mg/kg BW; PC, DOCA-salt with the administration of lisinopril at a dose 15 mg/kg BW; HE, DOCA-salt with the administration of black garlic extract at a dose of 100 mg/kg BW; HN, DOCA-salt with the administration of black garlic nanoemulsion at a dose of 100 mg/kg BW; LE, DOCA-salt with the administration of black garlic extract at a dose of 50 mg/kg BW; LN, DOCA-salt with the administration of black garlic nanoemulsion at a dose of 50 mg/kg BW. Values with different capital letters (A–D) in the same column are significantly different (*p* < 0.05). TNF-α, tumor necrosis factor-α; IL-6, interleukin-6; IL-1β, interleukin-1β; AChE, acetylcholinesterase; SOD, superoxide dismutase; CAT, catalase; GSH, glutathione; GSH-Px, glutathione peroxidase; MDA, malondialdehyde.

## Data Availability

Data is contained within the article or Supplementary Material.

## Supplementary Materials

The following are available online at https://www.mdpi.com/article/10.3390/antiox10101611/s1.
