# Supplementary material for: Effects of Black Garlic Extract and Nanoemulsion on the Deoxy Corticosterone Acetate-Salt Induced Hypertension and Its Associated Mild Cognitive Impairment in Rats
Source: Antioxidants (Basel). 2021 Oct 13;10(10):1611. doi: 10.3390/antiox10101611 (PMC8533483; doi:10.3390/antiox10101611)
Supplement: Supplementary file 1 [file antioxidants-10-01611-s001.zip › antioxidants-1377014-supplementary.pdf]

Supplementary Information

for

Effects of black garlic extract and nanoemulsion on the deoxy corticosterone acetate-salt induced hypertension and its associated mild cognitive impairment in rats.

Chun-Yu Chen[1,†], Tsung-Yu Tsai[1,†] and Bing-Huei Chen[1,2,*]

[1]Department of Food Science, Fu Jen Catholic University, New Taipei City 242, Taiwan.

[2]Department of Nutrition, China Medical University, Taichung 404, Taiwan

[†]Equally contributed to this article

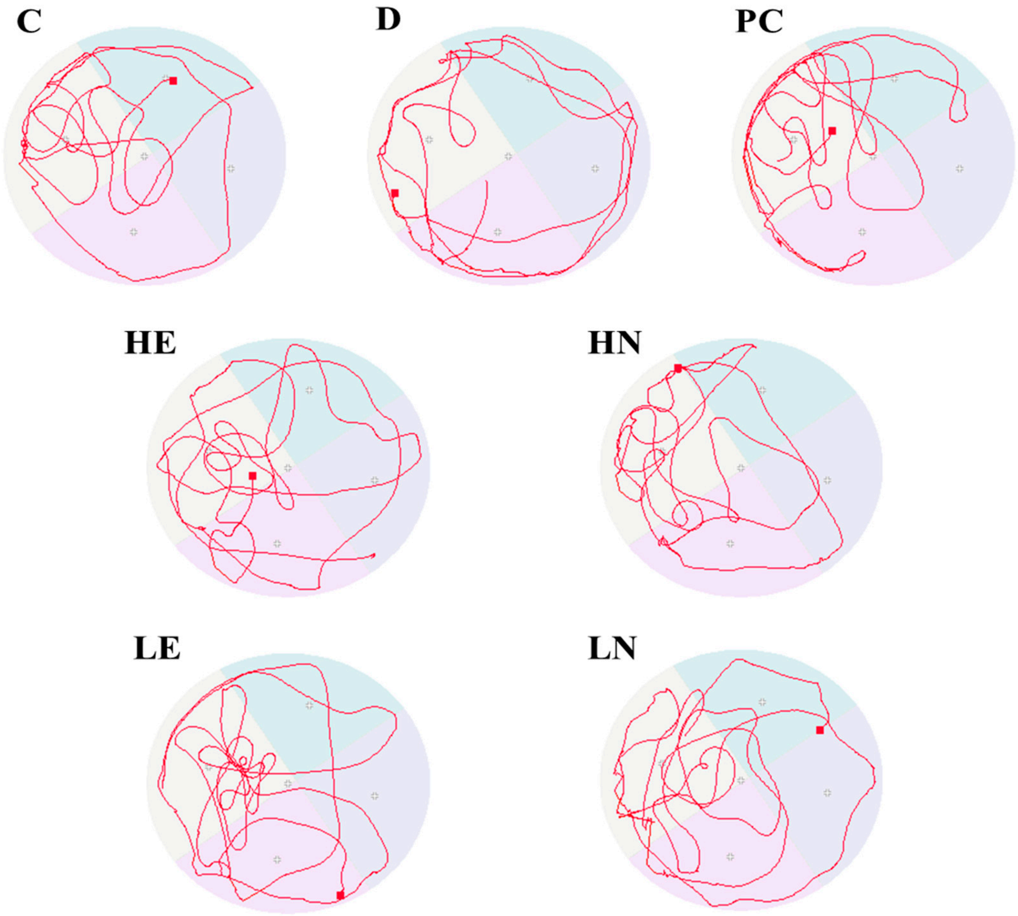

Figure S1. Effect of administration of black garlic extract and nanoemulsion on swimming pathways in Morris water maze in DOCA-salt induced hypertension and its associated mild cognitive impairment in rats. Data are presented as means ± standard deviation (n=6); C, control; D, DOCA-salt at a dose 25 mg/kg BW; PC, DOCA-salt with administration of lisinopril at a dose 15 mg/kg BW; HE, DOCA-salt with administration of black garlic extract at a dose of 100 mg/kg BW; HN, DOCA-salt with administration of black garlic nanoemulsion at a dose of 100 mg/kg BW; LE, DOCA-salt with administration of black garlic extract at a dose of 50 mg/kg BW; LN, DOCA-salt with administration of black garlic nanoemulsion at a dose of 50 mg/kg BW.

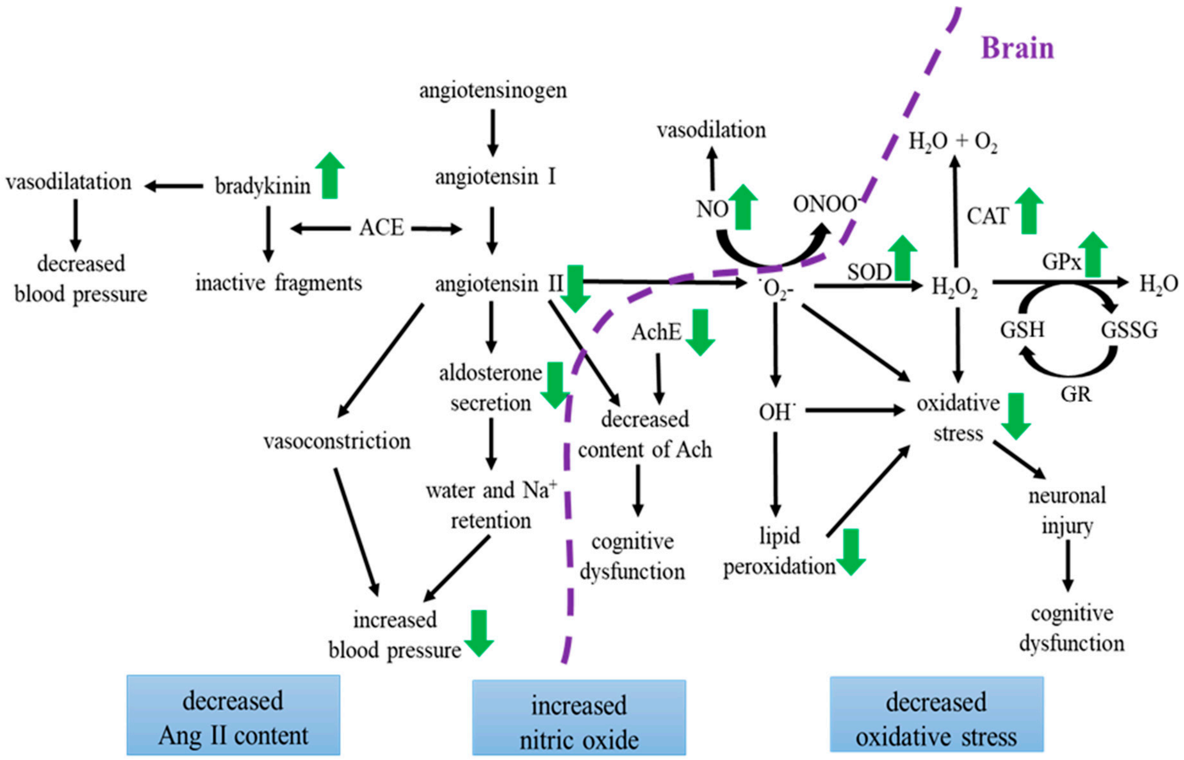

Figure S2. Proposed mechanism of black garlic extract and nanoemulsion to improve DOCA-salt induced hypertension and its associated mild cognitive impairment in rats.

Table S1. Particle size, polydispersity and zeta-potential of black garlic nanoemulsion during storage at 4°C for 90 days.

storage time (day)	particle size (nm)^{a}	polydispersity index^{a}	Zeta-potential (mV)^{a}
0	10.8 ± 0.3^{H}	0.135 ± 0.031^{AB}	-60.7 ± 2.2^{ABCD}
7	11.3 ± 0.4^{G}	0.160 ± 0.028^{A}	-62.6 ± 0.4^{AB}
14	12.8 ± 0.3^{BC}	0.136 ± 0.012^{AB}	-59.8 ± 2.3^{ABCDE}
21	11.6 ± 0.2^{FG}	0.123 ± 0.024^{BC}	-63.0 ± 2.5^{A}
28	11.8 ± 0.1^{EFG}	0.103 ± 0.014^{CDE}	-61.8 ± 0.6^{ABC}
35	12.3 ± 0.2^{CD}	0.116 ± 0.022^{BCD}	-60.7 ± 0.7^{ABCD}
42	12.1 ± 0.1^{DEF}	0.114 ± 0.013^{BCD}	-59.1 ± 2.1^{CDE}
49	12.3 ± 0.6^{CDE}	0.084 ± 0.006^{E}	-57.5 ± 3.3^{DEF}
56	11.8 ± 0.1^{EFG}	0.099 ± 0.009^{CDE}	-56.6 ± 1.6^{EF}
63	12.8 ± 0.3^{BC}	0.125 ± 0.007^{BC}	-57.0 ± 1.3^{EF}
70	12.9 ± 0.2^{B}	0.113 ± 0.005^{BCD}	-59.5 ± 1.3^{BCDE}
77	13.5 ± 0.2^{A}	0.095 ± 0.008^{DE}	-56.8 ± 1.4^{EF}
84	13.0 ± 0.5^{AB}	0.106 ± 0.012^{CDE}	-57.0 ± 1.3^{EF}
90	12.7 ± 0.4^{BC}	0.134 ± 0.016^{AB}	-55.6 ± 1.3^{F}

[a]Data shown are mean ± standard deviation (n = 3). Data with different capital letters (A-H) in the same column are significantly different at $p < 0.05$.

Table S2. Particle size, zeta-potential and polydispersity index changes as affected by black garlic nanoemulsion during heating at 40-100°C for varied time length (0.5-2.0 h)

Temperature (°C)	Particle size (nm)				Zeta-potential (mV)				Polydispersity index			
	0.5 h	1.0 h	1.5 h	2.0 h	0.5 h	1.0 h	1.5 h	2.0 h	0.5 h	1.0 h	1.5 h	2.0 h
Control (unheated)	11.2				-61.3				0.130			
40	12.5	12.3	13.2	13.2	-57.1	-50.5	-45.1	-41.2	0.129	0.132	0.127	0.141
60	13.7	13.1	13.7	13.5	-49.5	-40.1	-35.0	-30.6	0.136	0.132	0.129	0.134
80	12.4	13.1	13.0	14.1	-39.2	-27.1	-25.6	-21.4	0.119	0.144	0.141	0.153
100	13.3	13.5	13.6	13.4	-32.2	-26.8	-23.6	-18.1	0.133	0.178	0.193	0.164

Table S3. Effect of administration of black garlic extract and nanoemulsion on body weight in DOCA-salt induced hypertension and its associated mild cognitive impairment in rats.

group	body weight (g)						
	week 0	week 1	week 2	week 3	week 4	week 5	week 6
C	255 ± 9^{A}	300 ± 15^{A}	339 ± 18^{A}	371 ± 20^{A}	393 ± 25^{A}	421 ± 28^{A}	446 ± 31^{A}
D	256 ± 5^{A}	302 ±10^{A}	332 ± 14^{A}	349 ± 28^{A}	369 ± 18^{A}	385 ± 35^{A}	406 ± 28^{AB}
PC	255 ± 8^{A}	302 ± 14^{A}	331 ± 19^{A}	358 ± 16^{A}	373 ± 21^{A}	400 ± 28^{A}	414 ± 28^{AB}
HE	255 ± 9^{A}	297 ± 12^{A}	327 ± 16^{A}	356 ± 18^{A}	374 ± 21^{A}	396 ± 21^{A}	406 ± 29^{B}
HN	259 ± 8^{A}	303 ± 16^{A}	333 ± 24^{A}	368 ± 31^{A}	384 ± 30^{A}	410 ± 35^{A}	420 ± 33^{AB}
LE	256 ± 4^{A}	307 ± 10^{A}	340 ± 11^{A}	376 ± 18^{A}	395 ± 14^{A}	415 ± 25^{A}	428 ± 23^{AB}
LN	256 ± 6^{A}	297 ± 14^{A}	324 ± 21^{A}	352 ± 24^{A}	374 ± 29^{A}	391 ± 29^{A}	405 ± 31^{B}
	week 7	week 8	week 9	week 10	week 11	week 12	week 13
C	467 ± 32^{A}	488 ± 33^{A}	504 ± 31^{A}	512 ± 35^{A}	525 ± 33^{A}	536 ± 37^{A}	547 ± 34^{A}
D	420 ± 27^{A}	432 ± 24^{B}	444 ± 27^{B}	448 ± 29^{B}	471 ± 23^{AB}	470 ± 34^{B}	484 ± 31^{B}
PC	436 ± 34^{A}	443 ± 36^{B}	461 ± 33^{B}	467 ± 37^{AB}	470 ± 40^{B}	485 ± 42^{B}	489 ± 39^{B}
HE	427 ± 32^{A}	430 ± 33^{B}	451 ± 36^{B}	464 ± 32^{B}	470 ± 29^{B}	474 ± 26^{B}	489 ± 30^{B}
HN	447 ± 29^{A}	450 ± 31^{B}	462 ± 38^{B}	474 ± 33^{AB}	487 ± 31^{AB}	496 ± 27^{AB}	497 ± 21^{B}
LE	437 ± 20^{A}	441 ± 27^{B}	456 ± 31^{B}	475 ± 29^{AB}	486 ± 26^{AB}	491 ± 31^{B}	502 ± 31^{B}
LN	429 ± 27^{A}	435 ± 29^{B}	453 ± 34^{B}	464 ± 36^{B}	470 ± 40^{B}	477 ± 36^{B}	481 ± 34^{B}

Data are presented as means ± standard deviation (n=6); C, control; D, DOCA-salt at a dose 25 mg/kg BW; PC, DOCA-salt with administration of lisinopril at a dose 15 mg/kg BW; HE, DOCA-salt with administration of black garlic extract at a dose of 100 mg/kg BW; HN, DOCA-salt with administration of black garlic nanoemulsion at a dose of 100 mg/kg BW; LE, DOCA-salt with administration of black garlic extract at a dose of 50 mg/kg BW; LN, DOCA-salt with administration of black garlic nanoemulsion at a dose of 50 mg/kg BW. Values with different capital letters (A-B) in the same column were significantly different ($p < 0.05$).

Table S4. Effect of administration of black garlic extract and nanoemulsion on daily water intake in DOCA-salt induced hypertension and its associated mild cognitive impairment in rats

group	daily water intake (mL)						
	week 0	week 1	week 2	week 3	week 4	week 5	week 6
C	52 ± 6^{A}	48 ± 5^{B}	53 ± 7^{B}	54 ± 7^{B}	53 ± 7^{C}	53 ± 10^{B}	50 ± 4^{B}
D	50 ± 9^{A}	100 ± 21^{A}	150 ± 48^{A}	208 ± 37^{A}	233 ± 79^{A}	218 ± 65^{A}	203 ± 51^{A}
PC	48 ± 10^{A}	87 ± 25^{A}	136 ± 40^{A}	160 ± 29^{A}	173 ± 47^{AB}	200 ± 64^{A}	190 ± 30^{A}
HE	51 ± 4^{A}	95 ± 24^{A}	138 ± 47^{A}	169 ± 43^{A}	166 ± 43^{B}	198 ± 58^{A}	185 ± 29^{A}
HN	56 ± 7^{A}	96 ± 43^{A}	151 ± 49^{A}	188 ± 70^{A}	208 ± 57^{AB}	192 ± 80^{A}	183 ± 46^{A}
LE	54 ± 6^{A}	91 ± 19^{A}	159 ± 60^{A}	169 ± 46^{A}	222 ± 45^{AB}	238 ± 77^{A}	202 ± 93^{A}
LN	55 ± 8^{A}	98 ± 27^{A}	148 ± 59^{A}	181 ± 62^{A}	192 ± 73^{AB}	192 ± 51^{A}	200 ± 52^{A}
	week 7	week 8	week 9	week 10	week 11	week 12	week 13
C	54 ± 12^{B}	55 ± 11^{B}	54 ± 8^{B}	51 ± 12^{B}	61 ± 10^{B}	56 ± 5^{B}	56 ± 6^{B}
D	234 ± 65^{A}	182 ± 30^{A}	201 ± 42^{A}	200 ± 49^{A}	212 ± 37^{A}	179 ± 54^{A}	181 ± 23^{A}
PC	193 ± 37^{A}	190 ± 38^{A}	201 ± 41^{A}	189 ± 25^{A}	198 ±45^{A}	191 ± 46^{A}	201 ± 69^{A}
HE	233 ± 37^{A}	202 ± 39^{A}	198 ± 54^{A}	188 ± 47^{A}	189 ± 64^{A}	172 ± 29^{A}	193 ± 52^{A}
HN	199 ± 65^{A}	192 ± 54^{A}	183 ± 31^{A}	184 ± 58^{A}	192 ± 64^{A}	179 ± 33^{A}	204 ± 58^{A}
LE	240 ± 103^{A}	185 ± 107^{A}	191 ± 84^{A}	205 ± 59^{A}	187 ± 65^{A}	182 ± 84^{A}	192 ± 57^{A}
LN	195 ± 92^{A}	201 ± 49^{A}	206 ± 67^{A}	194 ± 45^{A}	182 ± 41^{A}	180 ± 43^{A}	195 ± 49^{A}

Data are presented as means ± standard deviation (n=6); C, control; D, DOCA-salt at a dose 25 mg/kg BW; PC, DOCA-salt with administration of lisinopril at a dose 15 mg/kg BW; HE, DOCA-salt with administration of black garlic extract at a dose of 100 mg/kg BW; HN, DOCA-salt with administration of black garlic nanoemulsion at a dose of 100 mg/kg BW; LE, DOCA-salt with administration of black garlic extract at a dose of 50 mg/kg BW; LN, DOCA-salt with administration of black garlic nanoemulsion at a dose of 50 mg/kg BW. Values with different capital letters (A-B) in the same column were significantly different ($p < 0.05$).

Table S5. Effect of administration of black garlic extract and nanoemulsion on the probe test in Morris water maze in DOCA-salt induced hypertension and its associated mild cognitive impairment in rats.

groups	time in target quadrant (s)	average of other 3 quadrants (s)
C	41.66 ± 6.89^{A}	15.58 ± 2.30^{B}
D	25.51 ± 6.46^{B}	21.17 ± 1.70^{A}
PC	40.72 ± 10.59^{A}	15.76 ± 3.81^{B}
HE	37.73 ± 7.41^{A}	16.99 ± 2.26^{B}
HN	37.97 ± 8.71^{A}	16.90 ± 2.58^{B}
LE	30.68 ± 11.85^{AB}	18.31 ± 3.00^{AB}
LN	33.39 ± 8.71^{AB}	18.51 ± 2.90^{AB}

Data are presented as means ± standard deviation (n=6); C, control; D, DOCA-salt at a dose 25 mg/kg BW; PC, DOCA-salt with administration of lisinopril at a dose 15 mg/kg BW; HE, DOCA-salt with administration of black garlic extract at a dose of 100 mg/kg BW; HN, DOCA-salt with administration of black garlic nanoemulsion at a dose of 100 mg/kg BW; LE, DOCA-salt with administration of black garlic extract at a dose of 50 mg/kg BW; LN, DOCA-salt with administration of black garlic nanoemulsion at a dose of 50 mg/kg BW. Values with different capital letter (A-B) in the same column were significantly different ($p < 0.05$).
